# Antimicrobial Susceptibility and Association with Toxin Determinants in *Clostridium perfringens* Isolates from Chickens

**DOI:** 10.3390/microorganisms8111825

**Published:** 2020-11-19

**Authors:** Bai Wei, Se-Yeoun Cha, Jun-Feng Zhang, Ke Shang, Hae-Chul Park, JeongWoo Kang, Kwang-Jick Lee, Min Kang, Hyung-Kwan Jang

**Affiliations:** 1 Center for Poultry Diseases Control, Department of Veterinary Infectious Diseases and Avian Diseases, College of Veterinary Medicine and Jeonbuk National University, Iksan 54596, Korea; weibai116@hotmail.com (B.W.); kshmnk@hanmail.net (S.-Y.C.); jfzhang018@gmail.com (J.-F.Z.) shangke0624@gmail.com (K.S.); 2 Veterinary Drugs & Biologics Division, Animal and Plant Quarantine Agency (QIA), Gimcheon 39660, Korea; sungpark@korea.kr (H.-C.P.); hijach@korea.kr (J.K.); leekwj@korea.kr (K.-J.L.)

**Keywords:** *C. perfringens*, chicken, antimicrobials resistance, growth promoters, virulence factors

## Abstract

The aim of the present study was to investigate variation in antimicrobial resistance in *Clostridium perfringens* (*C. perfringens*) isolated from chickens after withdrawal of antimicrobial growth promoters (AGPs); and to investigate the correlation between the presence of toxin genes (*cpb2*, *netB*, and *tpeL*) and antimicrobial resistance. Altogether, 162 isolates of *C. perfringens* were obtained from chickens displaying clinical signs of necrotic enteritis (*n* = 65) and from healthy chickens (*n* = 97) in Korea during 2010–2016. Compared to before AGP withdrawal, increased antimicrobial resistance or MIC_50_/MIC_90_ value was observed for nine antimicrobials including penicillin, tetracycline, tylosin, erythromycin, florfenicol, enrofloxacin, monensin, salinomycin, and maduramycin. Significantly (*p* < 0.05) higher resistance to gentamicin, clindamycin, and virginiamycin was found in isolates from chickens with necrotic enteritis compared to those from healthy chickens. *tpeL* gene was not detected in *C. perfringens* isolates from healthy chickens. A correlation between toxin gene prevalence and antibiotic resistance was found in the *C. perfringens* isolates. Because the usage of antimicrobials may contribute to the selection of both resistance and toxin genes, these can potentially make it challenging to control antimicrobial resistance in pathogenic colonies. Therefore, a more complete understanding of the interplay between resistance and virulence genes is required.

## 1. Introduction

*Clostridium perfringens* is a Gram-positive, anaerobic spore-forming bacterium that causes a wide variety of diseases in humans and animals and is ubiquitous in the environment including wastewater, dust, air, and healthy human and animal intestinal tracts [1]. Necrotic enteritis (NE) caused by *C. perfringens* has been one of the most economically important and financially crippling enteric poultry diseases in broiler chickens around the world [1]. From a financial point of view, the cost of subclinical NE can be as high as five cents per bird, and NE outbreaks can cost the global broiler industry nearly two billion US dollars every year [2]. Historically, *C. perfringens* isolates are categorized into the five toxin types A, B, C, D, and E, depending on the production of four major toxins including alpha (α), beta (β), epsilon (ε), and iota (ι). *C. perfringens* type A is the most common toxin type isolated from chickens suffering from NE [1]. Recently, based on a new introduced toxin-based typing system, *C. perfringens* type F consists of isolates that produce *C. perfringens* enterotoxin (CPE), type G isolates that produce NetB toxin (encoded by the gene *netB*) which has been found to be a critical virulence factor in the pathogenicity of NE in chickens [3]. In addition to these toxin genes, *tpeL*, has recently been identified in some chickens with NE -derived *C. perfringens* [4]. Following its first description, the gene *cpb2* has been found in *C. perfringens* isolates from a variety of animals suffering from enteric disease [5].

In most modern broiler-producing countries including Korea, antimicrobial growth promoters (AGPs) have been largely used during poultry production. Considering the emergence and spread of antimicrobial resistance, Korea prohibited the addition of AGPs to animal feeds since July 2011; while anticoccidial drugs including monensin, salinomycin, and maduramycin were still used in feed additives in poultry [6,7]. Nevertheless, NE re-emerged in broiler chickens in Korea as a consequence of AGP withdrawal from feed, and a high use of antimicrobials to treat this disease in broilers has been reported [8]. In view of the above, and the fact that antimicrobial therapy is still the most effective measure to control NE, we investigated the effect of AGP withdrawal from feed on the susceptibility of *C. perfringens* to antimicrobials of relevance to poultry production.

Antimicrobial use is not only associated with increased antimicrobial resistance among bacterial pathogens, but selection due to antimicrobial use may also contribute to positive/negative correlation with virulence determinants [9]. Resistance and virulence may not always be independent properties, and their relationship may play an important role in the pathogenesis of *C. perfringens* infection [9,10,11]. There have been no studies demonstrating such a link between antimicrobial resistance and virulence determinants in *C. perfringens* populations. Therefore, the aim of the present study was to investigate the variation in antimicrobial resistance in *C. perfringens* isolated from chicken, especially after AGP withdrawal; we also aimed to investigate the correlation between the presence of toxin genes (*cpb2*, *netB*, and *tpeL*) and antimicrobial resistance in *C. perfringens* isolates.

## 2. Materials and Methods

### 2.1. Bacterial Isolates

This study was a part of the research project “Development of therapeutic technique for using alternatives to antibiotics in necrotic enteritis of broiler chickens (PJ907105)” in Korea. Altogether, 162 isolates of *C. perfringens* were obtained from chickens displaying clinical signs of NE (*n* = 65) and from healthy chickens (*n* = 97) from 2010 to 2016 in Korea. Of these, 65 isolates (24 isolates, 2010–2011; 41 isolates, 2012–2016) of *C. perfringens* were collected from diseased chickens with NE submitted to our lab for necropsy examination. The chickens were presented with clinical signs including bloody or dark diarrhea, severe depression, and anorexia. The tissue samples were collected under the supervision of veterinarians. The collection of samples from chicken does not require an ethics statement and the owners provided informed consent for the research and publication of the results. After dissection, the gross lesions included multiple foci of mucosal necrosis on the lower part of the small intestine in diseased chickens. *C. perfringens* isolates were collected from the small intestine or liver with NE. For each farm, at least 3 to 4 colonies were collected for next molecular diagnosis, and one isolate was collected from a single chicken farm after final identification. Meanwhile, isolation of *C. perfringens* in healthy chickens was carried out from 2010. At the beginning of the study, no permission was required for disease monitoring and the animals could be used for research or teaching purposes. The owners provided informed consent for the clinical research and all the procedure under the supervision of veterinarians. The other 97 isolates (37 isolates, 2010–2011; 60 isolates, 2012–2016) were collected from the cecum or small intestine of clinically-healthy chickens obtained from 177 farms, and one isolate was obtained per farm. Primary isolates were checked visually for a typical *C. perfringens* double-hemolysis zone surrounding the colonies on sheep’s blood agar (Komed, Seongnam, South Korea) and then the *C. perfringens* isolates were confirmed by PCR reaction to detect the *cpa* gene [12]. Isolates were preserved in preservation media with Brain heart infusion (BHI, BD Becton Dickinson, Franklin Lakes, MD, USA) and 20% glycerol, and kept at −70 °C until use.

### 2.2. Antimicrobial Susceptibility Test

Minimal inhibitory concentrations (MICs) were determined on Mueller Hinton agar plates (Oxoid Ltd., Basingstoke, England, UK) containing doubling dilutions of the antimicrobials including penicillin, ampicillin, amoxicillin, gentamicin, spectinomycin, tetracycline, tylosin, erythromycin, florfenicol, bacitracin, enrofloxacin, clindamycin, virginiamycin, monensin, salinomycin, maduramycin, and trimethoprim-sulfamethoxazole, which are used for *C. perfringens* and/or coccidiosis infection and are widely used in poultry industry (Table 1). *C. perfringens* ATCC 13124 was used as the quality control strain and MIC was read as the lowest antimicrobial concentration inhibiting visible bacterial growth after 18–24 h of anaerobic incubation. The MIC_50_ and MIC_90_ represent the MIC value at which ≥50% and ≥90%, respectively, of the isolates within a test population were inhibited.

Standardized guidelines to interpret MIC values for *C. perfringens* are not well established. Therefore, we used the interpretation criteria for gentamicin and trimethoprim-sulfamethoxazole available from the Clinical and Laboratory Standards Institute (CLSI) for antimicrobial susceptibility testing of anaerobic bacteria [13], and those for penicillin, ampicillin, clindamycin [14], amoxicillin [15], enrofloxacin, tetracycline, florfenicol, bacitracin, erythromycin, and virginiamycin from published literature [16,17].

### 2.3. Toxin Gene Detection

All *C. perfringens* isolates were tested for the presence of *cpa*, *cpb*, *iA*, *etx*, *cpb2*, and *cpe* which respectively encode alpha toxin, beta toxin, iota toxin, epsilon toxin, beta2 toxin, and enterotoxin of *C. perfringens*, based on a procedure described previously [12]. *C. perfringens* ATCC 13124 was as positive control for *cpa* used in this study. The presence of the *netB* and *tpeL* genes was detected based on a procedure described previously [18]. Electrophoresis was performed on a 1% agarose gel with ethidium bromide using standard procedures.

### 2.4. Statistical analyses

Data management and all statistical analyses were done using the software SPSS version 19.0 (IBM, Armonk, NY, USA). The Chi-Square test was used to compare groups for the difference between antimicrobial resistance and prevalence of toxin genes. Association between various antimicrobial resistance outcomes (resistance or susceptibility) and presence of the toxin genes was determined via calculation of odds ratios (OR) with 95% confidence intervals (CI). An OR of >1 indicated that toxin gene prevalence was positively associated with resistance, and an OR of <1 indicated a negative association. All statistical significance tests were done using a cut-off value of 5%.

## 3. Results

### 3.1. Antimicrobial Susceptibility Testing

The distribution of MICs of the 17 antimicrobial agents tested against the 162 *C. perfringens* isolates from chickens is shown in Table 1 and Figure S1. High prevalence of resistance was observed against gentamicin (93.2%), virginiamycin (82.1%), trimethoprim-sulfamethoxazole (77.8%), and tetracycline (60.5%). Moreover, resistance to enrofloxacin (43.8%), clindamycin (38.3%), erythromycin (22.8%), bacitracin (17.3%), penicillin (1.2%), and florfenicol (0.6%) was also found. All isolates showed sensitivity to ampicillin and amoxicillin and showed a low MIC of these two antimicrobials. The MIC_50_/MIC_90_ ratios for monensin, salinomycin, and maduramycin were 1/2, 0.25/0.25, and 0.25/0.5 μg/mL, respectively.

Compared to *C. perfringens* isolates from 2010–2011, those from 2012–2016 were more likely to be resistant to most of the antimicrobials tested in the present study. A significantly (*p* < 0.05) higher percentage of resistance to enrofloxacin was found in *C. perfringens* isolates from 2012–2016. Further, the MIC_50_ and/or MIC_90_ values of penicillin, tetracycline, tylosin, erythromycin, florfenicol, enrofloxacin, monensin, salinomycin, and maduramycin against the 2012–2016 isolates were found to be higher than those against the 2010–2011 isolates. A significantly (*p* < 0.05) lower percentage of resistance to gentamicin, clindamycin, and virginiamycin was found in the 2012–2016 isolates than in the 2010–2011 isolates.

The prevalence of antimicrobial resistance in *C. perfringens* isolates from healthy chickens and chickens with NE differed against five antimicrobials (Table 2). Significantly (*p* < 0.05) higher resistance to gentamicin, clindamycin, and virginiamycin but significantly (*p* < 0.05) lower resistance to enrofloxacin was found in *C. perfringens* from chickens with NE compared with healthy chickens. There was no difference (*p* > 0.05) in antimicrobial resistance to the other five antimicrobials including penicillin, tetracycline, erythromycin, bacitracin, clindamycin, and trimethoprim-sulfamethoxazole between the isolates from healthy chickens and chickens with NE.

### 3.2. Toxin Gene Prevalence in C. perfringens Isolates

Results of PCR assays used for toxinotyping of *C. perfringens* showed that all 162 isolates were found to have only the *cpa* except for *netB* gene. Among these, 38 isolates with *netB* gene were *C. perfringens* type G and other isolates are type A. A total of 126 (77.8%), 38 (23.5%), and eight (4.9%) isolates were positive for *cpb2*, *netB*, and *tpeL* genes, respectively, in PCR assays (Table 3). The *cpb2* and *netB* genes were both detected in *C. perfringens* isolates from healthy chickens and chickens with NE, and the *tpeL* gene was only detected in *C. perfringens* isolates from chickens with NE. Significantly (*p* < 0.05) higher prevalence of the *cpb2*, *netB*, and *tpeL* genes was found in *C. perfringens* isolates from chickens with NE (87.7%, 36.9%, and 12.3%) than in the isolates from healthy chickens (71.1, 14.4, and 0%).

A total of four toxin gene patterns including the presence of *cpb2/netB/tpeL*, *cpb2/netB*, *cpb2*, or *netB* were found in the 162 *C. perfringens* isolates (Table 3), and the toxin gene pattern *cpb2/netB/tpeL* was only found in the isolates from chickens with NE. A total of 34 isolates with no toxin genes (*cpb2*, *netB*, and *tpeL*) was detected in the present study, and a significantly (*p* < 0.05) higher prevalence of these isolates was found in healthy chickens (27.8%) than in chickens with NE (10.8%).

### 3.3. Associations between Antimicrobial Resistance Outcomes and the Presence of Toxin Genes

The relationship between antimicrobial resistance and toxin gene carriage is shown in Table 4. Carriage of *cpb2* was more common among isolates resistant to gentamicin (OR, 2.222; 95% CI, 1.013–4.874; *p* = 0.001), tetracycline (OR, 1.512; 95% CI, 1.222–1.872; *p* < 0.001), or trimethoprim-sulfamethoxazole (OR, 1.351; 95% CI, 1.028–1.774; *p* = 0.006), while being more common among bacitracin-susceptible isolates (OR, 0.696; 95% CI, 0.5–0.969; *p* = 0.004). The *netB* gene was more common among virginiamycin-resistant isolates (OR, 8.068; 95% CI, 1.153–56.436; *p* = 0.005) than virginiamycin-susceptible isolates, while being more common among those susceptible to tetracycline (OR, 0.266; 95% CI, 0.142–0.498; *p* < 0.001), erythromycin (OR, 0.29; 95% CI, 0.094–0.888; *p* = 0.012), enrofloxacin (OR, 0.458; 95% CI, 0.239–0.879; *p* = 0.013), or clindamycin (OR, 0.501; 95% CI, 0.254–0.986; *p* = 0.034) than in the corresponding resistant isolates. The *tpeL* gene was more common among isolates susceptible to tetracycline (OR, 0.218, 95% CI, 0.045–1.045; *p* = 0.035) or enrofloxacin (OR, 0.142; 95% CI, 0.018–1.092; *p* = 0.035) than in the corresponding resistant isolates.

## 4. Discussion

In the present study, we investigated antimicrobial resistance in *C. perfringens* isolates from chickens in Korea. Among all the tested antimicrobials, penicillin, ampicillin, amoxicillin, florfenicol, monensin, salinomycin, and maduramycin had relatively low MIC values, in agreement with studies conducted in China, Canada, and the United States which reported that these antimicrobials were effective in treating NE in chickens [16,19,20]. We also investigated the antimicrobial resistance of *C. perfringens* isolates from chickens before and after AGP withdrawal in Korea. It should be emphasized that AGP withdrawal from feed did not result in a comprehensive decrease in resistance; a higher level of resistance to nine antimicrobials as reflected in their MIC_50_ and/or MIC_90_ values (penicillin, tetracycline, tylosin, erythromycin, florfenicol, enrofloxacin, monensin, salinomycin, and maduramycin) was found in *C. perfringens* isolates obtained after AGP withdrawal compared with those obtained before (Table 1). These results most likely reflect the fact that rather than antimicrobials supplied in animal feed, much larger amounts of antimicrobials had been used for disease prophylaxis and treatment in poultry production [21]. Remarkably, the total sale of antimicrobials in Korea decreased to its lowest level in 2013, while it increased again from 2014 (Animal and Plant Quarantine Agency, 2017). Therefore, the termination of the use of AGPs in animal feed might not significantly influence antimicrobial resistance over the several subsequent years (Tables S1 and S2), and co-selection due to other antimicrobials or resistant strains with low/no fitness cost will promote the persistence of resistance in farm animals [22,23,24].

Compared to the antimicrobial resistance in *C. perfringens* from healthy chickens, significantly higher resistance to gentamicin, clindamycin, and virginiamycin was found in chickens with NE. Among these three antimicrobials, virginiamycin is the one considered for control of *C. perfringens* in chickens [16]. Our finding is in agreement with previous studies showing that chickens with NE have a higher probability of being exposed to antimicrobial therapy than clinically healthy chickens, and that *C. perfringens* isolated from diseased chickens have a higher probability of developing resistance [20]. Therefore, the *C. perfringens* isolates with higher resistance found in chickens with NE suggest that a more cautious approach is required in selecting proper antimicrobials for disease treatment, and to prevent emergence and dissemination of resistant *C. perfringens*.

All of the isolates in the present study were classified as *C. perfringens* type A and G, and this finding was consistent with those reported previously in Korea and other countries, which indicated that type A and G is prevalent both in healthy and diseased chickens [1,3,18]. It has recently been reported that a high frequency of toxin genes was found in *C. perfringens* isolates from chickens with NE than in those from healthy chickens [25]. Our finding was also consistent with the above report, with all three toxin genes tested in this study showing high prevalence in NE -derived *C. perfringens* isolates. We also showed that 27.8% of healthy chicken isolates did not carry any toxin genes, which were significantly (*p* < 0.05) more than the corresponding NE-derived isolates (10.8%). This strongly supports a correlation between the presence of these toxin genes and NE, and the fact that these toxin genes play an important role in the pathogenesis of NE in chickens [1,25]. Further, the toxin gene *tpeL* and the toxin gene pattern *cpb2/netB/tpeL* were only found in isolates from chickens with NE and not in the healthy chicken isolates. This finding strongly indicated the involvement of the toxin gene *tpeL* in pathogenesis of NE in chickens as previously described [4]. The *tpeL* gene may potentiate the effect of other virulence attributes of NE -derived *C. perfringens* isolates as previously reported [4,26], and all the isolates from chickens with NE carrying the *tpeL* gene were found to co-carry *cpb2/netB* in the present study. As the contribution of *cpb2* and *tpeL* toxin genes in the pathogenesis of NE in chickens is yet to be established. Therefore, the contribution of *cpb2, tpeL, tpeL,* and *cpb2/netB* to the pathogenesis of NE needs to be evaluated further.

Our analysis of a large population of *C. perfringens* isolates from chickens provides epidemiological evidence for the association of antimicrobial resistance and the presence of toxin genes. First, *netB* gene prevalence had lower odds of association with resistance to tetracycline, erythromycin, enrofloxacin, or clindamycin in *C. perfringens* isolates, and *cpb2* had lower odds of association with bacitracin resistance. A negative correlation between the prevalence of virulence genes and antimicrobial resistance has been reported in *E. coli*, with quinolone resistance being negatively correlated with the prevalence of virulence genes [27]. This may be explained by the hypothesis that mutations in topoisomerase genes produce a reduction in DNA supercoiling that might have implications in the expression of some virulence genes [27]. Further, the resistance and toxin genes might be located on different incompatible plasmid groups, and co-existence of resistance and toxin genes in a strain may be uncommon [28]. Second, a positive correlation between the prevalence of toxin genes and antimicrobial resistance was found; *netB* gene prevalence had higher odds of association with *C. perfringens* isolates with virginiamycin resistance, and *cpb2* had higher odds of association with isolates resistant to gentamicin, tetracycline, or trimethoprim-sulfamethoxazole. Vilei et al. [10] have demonstrated that aminoglycoside treatment induces the expression of the *cpb2* gene in *C. perfringens* isolates obtained from horses, and that the treatment lead to a more accentuated and fatal progression of equine typhlocolitis [10]. These data provide evidence that antimicrobial selection pressure may not only result in antimicrobial resistance, but may also be involved in virulence gene expression and disease symptom status [9,29]. The continuous use of antimicrobials in animals may facilitate the spread of virulence genes within bacterial communities, and the acquisition of resistance and virulence determinants at the same time may represent a survival advantage to the microorganisms. In our study, the positive association of *netB* gene prevalence with virginiamycin resistance was observed; because the *netB* gene is a critical factor in NE development, high virginiamycin resistance and persistent usage of virginiamycin in farm animals may promote resistance and maintenance of pathogenic colonies in farms. We found that the total *netB* gene prevalence in *C. perfringens* isolates in our study (23.5%) was higher than that in a previous report (14.9%) in Korea [18]; these data raise the possibility that *netB* gene prevalence will continually increase if virginiamycin is used without limitation in farm animals in the future. Similar to the common use of virginiamycin as an antimicrobial to treat poultry disease, tetracycline and trimethoprim-sulfamethoxazole have also been largely used in the poultry industry. Such large usage of antimicrobials in animals may also promote the dissemination of *cpb2* in *C. perfringens* strains, not limited to chickens but also to other animals, although there is currently no direct evidence to support such a speculation.

Although our results suggested a plausible ecological and biological link between antimicrobial resistance outcomes and toxin gene existence in *C. perfringens* isolates via epidemiological evidence, the link between resistance and virulence is complex due to the diversity of antimicrobial resistance genes, virulence factors, and bacterial species. Our ability to show the linkage between antimicrobial resistance and toxin genes has limitations; we did not investigate the resistance mechanisms for all the antimicrobials due to the limited information available on antimicrobial resistance mechanisms in *C. perfringens*. However, our study is the first step in understanding whether there is a connection between resistance and virulence. We also did not investigate the genetic linkage, if any, between virulence and resistance genes. More in-depth molecular studies on the interactions between antimicrobial resistance and virulence determinants are needed to fully understand the interplay between resistance and virulence genes in *C. perfringens*.

## 5. Conclusions

In conclusion, the present study generated data regarding antimicrobial resistance and distribution of toxin genes among *C. perfringens* isolates from healthy and diseased chickens before and after the AGP ban in Korea, and analyzed the association between antimicrobial resistance and toxin gene prevalence. Our results show widespread decrease in susceptibility to antimicrobials in *C. perfringens* isolates obtained after the AGP ban, likely as a consequence of an increase in antimicrobial usage. The unique susceptibility pattern demonstrated in this study indicates potential strategies for therapeutic candidate selection for disease control after the AGP ban. High prevalence of toxin genes were found in *C. perfringens* isolates from diseased chickens. The toxin gene *tpeL* was only found in *C. perfringens* isolates from diseased chickens, indicating that *tpeL* may contribute significantly to the pathogenesis of NE; all *tpeL*-positive isolates showed the presence of *cpb2* and *netB*, suggesting that *tpeL* may potentiate the effect of other toxin determinants contributing to the pathogenesis of NE. In addition, a correlation between toxin gene prevalence and antimicrobial resistance was observed in *C. perfringens* communities, with both positive and negative correlations between the presence of toxin genes and antimicrobial resistance. Because the usage of antimicrobials may contribute to the selection of both resistance and toxin genes, these can potentially make it challenging to control antimicrobial resistance in pathogenic colonies. Therefore, a more complete understanding of the interplay between resistance and virulence genes is required.

## Figures and Tables

**Table 1:** Minimal inhibitory concentrations (MICs) against *Clostridium perfringens* isolates from chickens from 2010 to 2016.

Antimicrobial	Range	Breakpoint (μg/mL)	Total (*n* = 162)		2010–2011 (*n* = 61)		2012–2016 (*n* = 101)		*p* Value ^a^
			MIC_50_/MIC_90_	Resistance No. (%)	MIC_50_/MIC_90_	Resistance No. (%)	MIC_50_/MIC_90_	Resistance No. (%)	
Penicillin	≤0.06 to 2	1	≤0.06/0.25	2 (1.2)	≤0.06/0.12	0	≤0.06/0.25	2 (2.0)	
Ampicillin	≤0.12 to 0.25	16	≤0.12/0.25	0	≤0.12/0.25	0	≤0.12/≤0.12	0	
Amoxicillin	≤0.25	16	≤0.25/≤0.25	0	≤0.25/≤0.25	0	≤0.25/≤0.25	0	
Gentamicin	4 to ≥16	16	≥16/≥16	151 (93.2)	≥16/≥16	61 (100.0)	≥16/≥16	90 (89.1)	0.007
Spectinomycin	8 to ≥128	-^b^	≥128/≥128	-^b^	≥128/≥128	-^b^	≥128/≥128	-^b^	-^b^
Tetracycline	0.25 to ≥16	4	4/≥16	98 (60.5)	4/8	32 (52.5)	8/≥16	66 (65.3)	
Tylosin	≤2 to ≥16	-^b^	≤2/≥16	-^b^	≤2/8	-^b^	≤2/16	-^b^	-^b^
Erythromycin	0.12 to ≥16	16	4/≥16	37 (22.8)	2/≥16	16 (26.2)	4/≥16	21 (20.8)	
Florfenicol	1 to 8	8	1/2	1 (0.6)	≤1/≤1	0	2/2	1 (1.0)	
Bacitracin	1 to 64	32	1/32	28 (17.3)	≤1/32	9 (14.8)	≤1/32	19 (18.8)	
Enrofloxacin	0.12 to ≥32	2	1/≥32	71 (43.8)	0.25/32	15 (24.6)	2/32	56 (55.4)	<0.001
Clindamycin	0.5 to ≥8	8	2/≥8	62 (38.3)	≥8/≥8	32 (52.5)	2/≥8	30 (29.7)	0.004
Virginiamycin	1 to 16	4	4/8	133 (82.1)	4/≥16	60 (98.4)	4/≥16	73 (72.3)	<0.001
Monensin	≤0.12 to 4	-^b^	1/2	-^b^	≤0.12/0.25	-^b^	1/2	-^b^	-^b^
Salinomycin	≤0.12 to 0.5	-^b^	0.25/0.25	-^b^	≤0.12/≤0.12	-^b^	0.25/0.25	-^b^	-^b^
Maduramycin	0.12 to 0.5	-^b^	0.25/0.5	-^b^	0.25/0.25	-^b^	0.25/0.5	-^b^	-^b^
Trimethoprim-sulfamethoxazole	0.5/9.5 to >4/76	>4/76	4/76/4/76	126 (77.8)	≥4/76/≥4/76	44 (72.1)	≥4/76/≥4/76	82 (81.2)	

^a^, The Chi-square test of independence was used to test the difference in antimicrobial resistance between *Clostridium perfringens* isolates from 2010–2011 and 2012–2016; *p* value > 0.05 is not shown. -^b^, not available.

**Table 2:** Antimicrobial resistance of *Clostridium perfringens* isolates from chickens with or without necrotic enteritis.

Antimicrobial	Antibiotic Resistance, *n*/(%)		*p* Value ^a^
	Enteritis (*n* = 65)	Health (*n* = 97)	
Penicillin	0	2 (2.1)	
Gentamicin	65 (100.0)	86 (88.7)	0.007
Tetracycline	33 (50.8)	65 (67.0)	
Erythromycin	19 (29.2)	18 (18.6)	
Bacitracin	12 (18.5)	16 (16.5)	
Enrofloxacin	18 (27.7)	53 (54.6)	<0.001
Clindamycin	35 (53.8)	27 (27.8)	0.004
Virginiamycin	64 (98.5)	69 (71.1)	<0.001
Trimethoprim-sulfamethoxazole	48 (73.8)	78 (80.4)	

^a^, The Chi-square test of independence was used to test the difference in antimicrobial resistance between *Clostridium perfringens* isolates from chickens with or without necrotic enteritis; *p* value > 0.05 is not shown.

**Table 3:** Toxin gene prevalence in *Clostridium perfringens* isolates from chickens with or without necrotic enteritis.

	No. of Isolates	No. (%) of Toxin Genes			Toxin Gene Pattern, *n*/(%)				
		*cpb2*	*netB*	*tpeL*	*cpb2/netB/tpeL*	*cpb2/netB*	*cpb2*	*netB*	No Toxin Gene
Total	162	126 (77.8)	38 (23.5)	8 (4.9)	8 (4.9)	28 (17.3)	90 (55.6)	2 (1.2)	34 (21.0)
Enteritis	65	57 (87.7)	24 (36.9)	8 (12.3)	8 (12.3)	15 (23.1)	34 (52.3)	1 (1.5)	7 (10.8)
Healthy	97	69 (71.1)	14 (14.4)	0 (0.0)	0	13 (13.4)	56 (57.7)	1 (1.0)	27 (27.8)
*p* value ^a^		0.013	0.001	0.001	0.001				0.009

^a^, The Chi-square test of independence was used to test the difference in toxin gene prevalence between *Clostridium perfringens* isolates from chickens with or without necrotic enteritis; *p* value > 0.05 is not shown.

**Table 4:** Associations between antimicrobial resistance outcomes and the presence of toxin genes in *C. perfringens* isolates from chickens.

Antimicrobial	% of the Isolates Positive for Toxin Genes ^a^											
	*cpb2*				*netB*				*tpeL*			
	R	S	OR (95% CI)	*p* Value ^b^	R	S	OR (95% CI)	*p* Value ^b^	R	S	OR (95% CI)	*p* Value ^b^
Gentamicin	80.8	36.4	2.222 (1.013–4.874)	0.001	25.2	0	3.314 (0.494–22.210)		5.3	0	0.765 (0.105–5.577)	
Tetracycline	89.8	59.4	1.512 (1.222–1.872)	<0.001	11.2	42.2	0.266 (0.142–0.498)	<0.001	2.0	9.4	0.218 (0.045–1.045)	0.035
Erythromycin	70.3	80.0	0.878 (0.7–1.102)		8.1	28.0	0.29 (0.094–0.888)	0.012	0	6.4	0.362 (0.047–2.768)	
Bacitracin	57.1	82.1	0.696 (0.5–0.969)	0.004	10.7	26.1	0.41 (0.136–1.24)		0	6	0.504 (0.066–3.827)	
Enrofloxacin	77.5	78.0	0.993 (0.841–1.172)		14.1	30.8	0.458 (0.239–0.879)	0.013	0.0	8.8	0.142 (0.018–1.092)	0.01
Clindamycin	79.0	77.0	1.026 (0.868–1.213)		14.5	29.0	0.501 (0.254–0.986)	0.034	3.2	6.0	0.538 (0.112–2.581)	
Virginiamycin	80.5	65.5	1.228 (0.931–1.62)		27.8	3.4	8.068 (1.153–56.436)	0.005	6.0	0	2.067 (0.272–15.716)	
Trimethoprim-sulfamethoxazole	82.5	61.1	1.351 (1.028–1.774)	0.006	25.4	16.7	1.524 (0.692–3.355)		3.2	11.1	0.286 (0.075–1.086)	

^a^, R, resistance; S, susceptible; OR, odd ratios; CI, confidence intervals. ^b^, The Chi-square test of independence was used to test the difference in toxin gene prevalence between *Clostridium perfringens* isolates with or without antibiotic resistance; *p* value > 0.05 is not shown.

## Supplementary Materials

The following are available online at https://www.mdpi.com/2076-2607/8/11/1825/s1, **Table S1**: Antimicrobials used in chickens from 2010 to 2016 in Korea. Table S2: Antimicrobials used in feed additives and disease treatment in chickens from 2010 to 2016 in Korea. Figure S1: MIC histograms for 162 *Clostridium perfringens* isolates from chickens against 17 antimicrobials (A-Q) from 2010 to 2016, isolates from 2010–2011 (black) and 2012–2016 (grey).
