# Supplementary material for: Antimicrobial Susceptibility and Association with Toxin Determinants in Clostridium perfringens Isolates from Chickens
Source: Microorganisms. 2020 Nov 19;8(11):1825. doi: 10.3390/microorganisms8111825 (PMC7699427; doi:10.3390/microorganisms8111825)
Supplement: Supplementary file 1 [file microorganisms-08-01825-s001.pdf]

Table S1. Antimicrobials used in chickens from 2010 to 2016 in Korea

Antimicrobials	Antimicrobial usage (kg) in chickens from 2010 to 2016 in Korea						
	2010	2011	2012	2013	2014	2015	2016
Penicillins	16,246	13,078	16,471	26,829	20,377	18,510	26,209
Aminoglycosides	4,519	4,141	4,306	3,598	3,356	3,628	3,004
Tetracyclines	28,638	20,750	23,843	15,310	12,283	16,445	15,068
Macrolides	2,817	2,358	2,298	1,348	2,024	2,730	2,402
Phenicols	8,857	11,237	12,267	9,281	9,021	13,035	15,421
Bacitracin	16,580	7,302	256	775	353	551	2,756
Enrofloxacin	26,684	37,706	34,613	32,403	19,106	25,267	26,022
Lincosamides	1,078	916	1,098	1,033	1,054	1,598	1,986
Virginiamycin	3,266	1,264	356	253	348	516	24
Monensin	2,038	2,213	3,214	64	1,960	705	35
Salinomycin	36,308	35,946	30,344	7,064	21,620	26,980	16,560
Maduramycin	253	2,224	1,135	1,035	443	531	795
Trimethoprim-sulfamethoxazole	2,686	3,348	2,854	729	1,541	2,001	2,437

Table S2. Antimicrobials used in feed additives and disease treatment in chickens from 2010 to 2016 in Korea

Antimicrobial usage		Antimicrobial usage (kg) in chickens from 2010 to 2016 in Korea						
		2010	2011	2012	2013	2014	2015	2016
Feed additives	Monensin	2,035	1,745	385	8	1,285	1,285	0
	Salinomycin	30,197	16,586	11,132	11,191	5,350	8,160	0
	Maduramycin	0	564	303	245	136	136	70
Disease treatment	Monensin	3	468	2,829	56	678	705	50
	Salinomycin	6,111	19,360	19,212	7,064	16,270	18,820	16,560
	Maduramycin	253	1,660	832	1,035	307	531	795

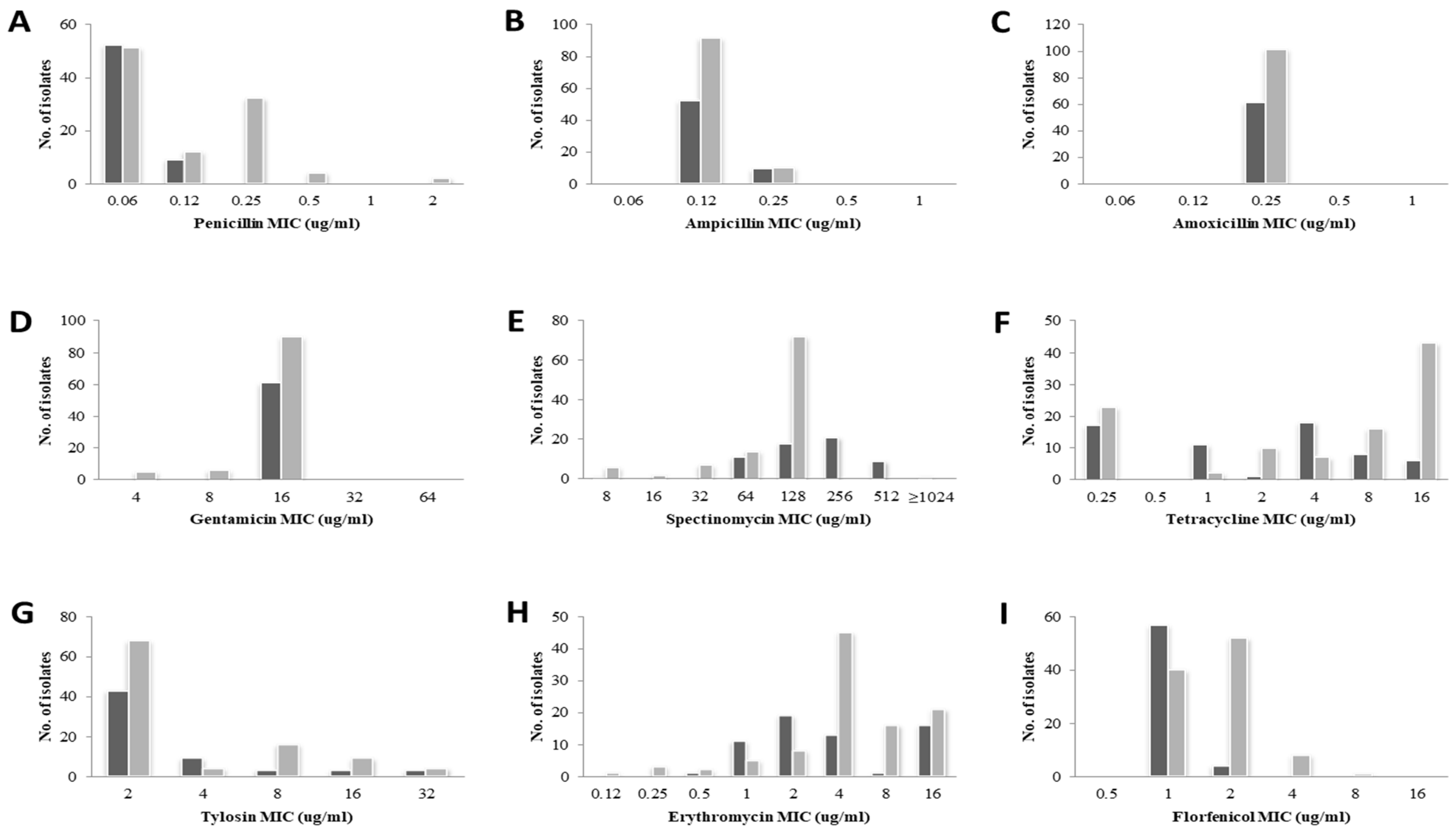
A
No. of isolates
Penicillin MIC (ug/ml)
0.06
0.12
0.25
0.5
1
2
B
Ampicillin MIC (ug/ml)
C
Amoxicillin MIC (ug/ml)
D
Gentamicin MIC (ug/ml)
4
8
16
32
64
E
Spectinomycin MIC (ug/ml)
128
256
512
≥1024
F
Tetracycline MIC (ug/ml)
G
Tylosin MIC (ug/ml)
H
Erythromycin MIC (ug/ml)
I
Florfenicol MIC (ug/ml)

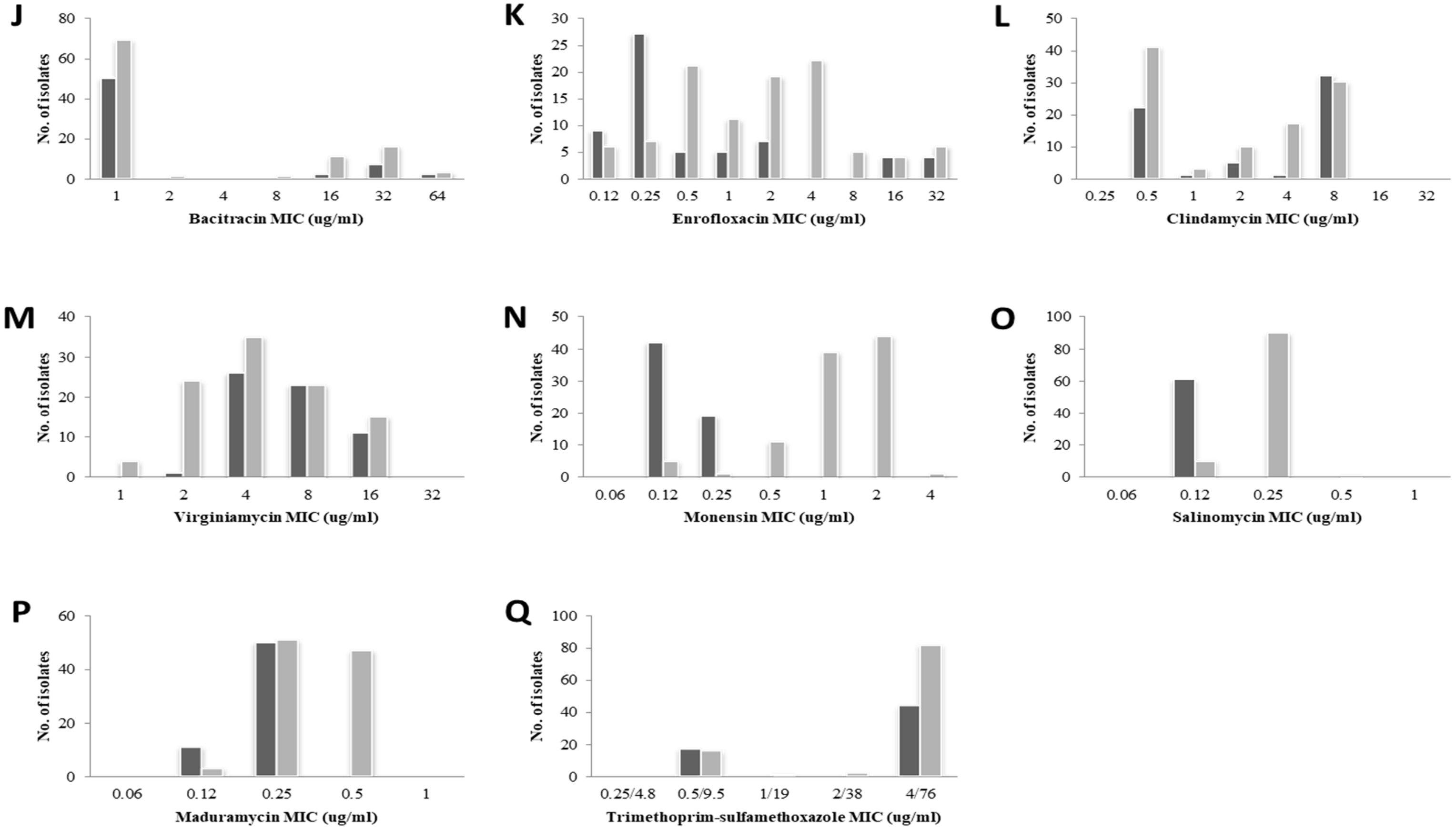

Fig. S1. MIC histograms for 162 *Clostridium perfringens* isolates from chickens against 17 antimicrobials (A-Q) from 2010 to 2016, isolates from 2010–2011 (black) and 2012–2016 (grey).
